# Evaluation of the Structural and Construct Validity of the Credibility and Expectancy Scale for Patients With Musculoskeletal Disorders

**DOI:** 10.7759/cureus.67639

**Published:** 2024-08-23

**Authors:** Hiroshi Takasaki, Yusuke Handa, Hiroki Chiba, Tomoya Kitamura

**Affiliations:** 1 Department of Physical Therapy, Saitama Prefectural University, Koshigaya, JPN; 2 Department of Rehabilitation, Minami Shinjuku Orthopedic Clinic, Tokyo, JPN; 3 Graduate School of Rehabilitation Science, Saitama Prefectural University, Koshigaya, JPN; 4 Department of Rehabilitation, Secomedic Hospital, Funabashi, JPN

**Keywords:** mckenzie method, mechanical diagnosis and therapy, musculoskeletal pain, psychometric, outcome, questionnaire, rationale, expectancy, credibility, treatment

## Abstract

Background

The Credibility Expectancy Questionnaire (CEQ) includes three items each on the credibility and expectancy subscales. Credibility indicates to what extent the treatment is reasonable, and expectancy indicates to what extent the treatment is expected to be effective. The CEQ has been assumed to have a two-factor structure: credibility and expectancy, among patients receiving psychotherapy. However, its internal structure has been unknown to patients receiving physical therapy for musculoskeletal disorders. This study aimed to explore the internal structure of the CEQ and preliminary investigate the construct validity of the CEQ among patients receiving physical therapy for musculoskeletal disorders.

Methodology

A multi-center prospective cohort study was conducted. Data from 100 patients receiving outpatient physical therapy for musculoskeletal disorders was collected using an anonymous paper-based survey. The initial survey was conducted immediately before the initial physical therapy session, and the second survey was conducted after the third to seventh physical therapy sessions. The Patient Specific Functional Scale 2.0 (PSFS 2.0) was collected in both surveys, and the CEQ and an 11-point global rating of change scale (GRCS) were collected in the second survey. Exploratory factor analysis was conducted for the CEQ, and internal consistency was assessed for each subscale and an identified factor structure. Convergent validity in construct validity was also assessed with the hypothesis that Pearson’s r values of each CEQ factor score to the PSFS 2.0 change scores and GRCS would range from 0.4 to 0.6.

Results

An exploratory factor analysis revealed a one-factor structure, where the percentage of the variance for the extraction sums of squared loadings was 62.8%. Cronbach’s alpha was 0.89 for all items, 0.91 for the credibility subscale, and 0.75 for the expectancy subscale. Hypothesized correlations to the PSFS 2.0 change score and GRCS were detected with the CEQ total score (r = 0.48 for the PSFS 2.0 change score and r = 0.59 for the GRCS) and each subscale score (credibility subscale, r = 0.48 for the PSFS 2.0 change score and r = 0.49 for the GRCS; and expectancy subscale, r = 0.43 for the PSFS 2.0 change score and r = 0.62 for the GRCS).

Conclusion

A single-factor internal structure of the CEQ was detected among patients receiving physical therapy for musculoskeletal disorders. Additionally, preliminary evidence of construct validity was detected with convergent validity between the CEQ and functional and perceived improvement.

## Introduction

Treatment credibility and expectancy have been less evaluated than treatment outcomes in musculoskeletal care to date. However, their importance is likely to be recognized in the future, as patient-centered care has been proposed to be a core element of optimal musculoskeletal care [1]. Patient treatment expectations have been shown to have some influence on treatment effectiveness [2,3].

Patient expectations have been defined and measured in many ways, necessitating appropriate terminology to be defined in the future. The expectation here is defined as the predicted treatment outcome expectation according to previous studies [4,5]. Among the measures to assess treatment expectation, one of the most widely recognized and nonspecific measures is the Credibility Expectancy Questionnaire (CEQ) [6]. For expectancy and credibility in the CEQ, treatment expectation here refers to “improvements that clients believe will be achieved on the basis of a particular treatment,” and treatment credibility is defined as “how believable, convincing, and logical the treatment is,” according to Kazdin [7]. Especially in the case of psychotherapy, such as cognitive behavioral therapy (CBT), treatment expectation and treatment credibility are considered to have different psychometric properties because what is felt by the mind and what is theoretically derived can be distinguished and used in the treatment. In fact, Devilly and Borkovec [6] analyzed the factor structure of the CEQ in patients undergoing psychotherapy, including CBT, and reported two-factor structures: credibility and expectancy. A similar two-factor structure, albeit with one item missing, was also reported in a study of the Portuguese version of the CEQ in patients undergoing psychotherapy [8]. However, the need for further validation of the factor structure of the CEQ is suggested, including the possibility of adding or deleting further questions [6].

The CEQ may be applicable not only to psychotherapy but also to the treatment of musculoskeletal disorders, as it has been used in previous and ongoing clinical trials for musculoskeletal disorders [9,10]. Nevertheless, to the best of the authors' knowledge, its factor structure has not been examined in patients with musculoskeletal disorders. Therefore, the primary aim of this study was to explore the internal structure of the CEQ among patients receiving physical therapy for musculoskeletal disorders. A secondary aim was to preliminary investigate the construct validity of the CEQ in those participants.

## Materials and methods

Design

A multi-center prospective cohort study was conducted at Minami Shinjuku Orthopedic Clinic and Secomedic Hospital, Japan. Data were collected using a paper-based survey and written consent was waived by submitting a complete set of questionnaires. The study was approved by the Saitama Prefectural University Research Ethics Committee (no. 22040).

Participants

Inclusion criteria include (1) >17 years of age with Japanese as their first language, (2) those receiving outpatient physical therapy for musculoskeletal disorders from physical therapists who were credential holders in the McKenzie Method® of Mechanical Diagnosis and Therapy® (MDT) in two medical institutions in Japan (Tokyo and Chiba), (3) those without diagnosis of neurological disorders or cognitive disorders, and (4) not pregnant. Exclusion criteria include those who did not come for physical therapy follow-up before the second survey and those with missing responses in the survey form.

Immediately before the initial physical therapy session, the following demographic and general data were collected to understand the characteristics of the participants: (1) age, (2) sex, (3) symptom location on a body chart with 22 distinct areas [11], (4) duration of the current episode of symptoms for which the physical therapy was referred. The symptom duration was the time since the last day when the patient did not feel any symptoms for more than one month and was categorized into three groups: less than seven days, eight days to three months, and greater than three months [12].

Data collection was continued until 100 analyzable data were obtained from July 2023 to July 2024. The sample size of 100 was determined as the COnsensus-based Standards for the selection of health Measurement Instruments (COSMIN) [13] requests seven times the number of items and ≥100 for very good quality studies assessing structural validity.

Physical therapists in charge of data collection

Physical therapy was provided by six physical therapists (mean (SD) of clinical experience = 13.7 (7.0) years). This was a cohort study and thus intervention was not controlled but followed the principles of the MDT. MDT is an individualized physical therapy approach that is based on a biopsychosocial framework maximizing patient education to promote patient self-management skills and minimizing the risk with considerations of mechanical load, and whose treatment principle is guided by MDT classifications [14].

Procedures and outcomes

At the initial physical therapy session, the participants completed a set of questionnaires that included the Patient Specific Functional Scale 2.0 (PSFS 2.0) [15,16]. The second survey was conducted after the third to seventh physical therapy sessions based on a previous study [17] and included; (1) the PSFS 2.0 with scores at the initial session; (2) an 11-point global rating of change scale (GRCS) [18,19], and (3) the CEQ [6], where the word “anxiety” was changed to “symptoms” in the original instructions, “We would like you to indicate below how much you believe, right now, that the therapy you are receiving will help to reduce your anxiety,” and the word “trauma symptoms” was changed to “symptoms” in the item descriptions in the Japanese version [20]. Participants who agreed to participate in this study submitted the anonymous second survey set in a box located in each institution, ensuring that their scores were blinded to their treatment physical therapist.

In the PSFS 2.0, each participant nominates up to three of the most important and challenging activities resulting from their musculoskeletal disorders. For each item, a score was given on an 11-point scale (0: no difficulty; 10: impossible to perform the activity). The difference between the first and second mean values was calculated and adjusted to show that positive values (-10 to 10) indicate improvement for the sake of simplicity. Such an individualized functional scale is considered to be more highly responsive than structured patient-reported outcome measures [21].

In the GRCS, patients rate their global perception of recovery on an 11-point scale (-5: very much worse; 0: unchanged; 5: completely recovered). GRCS is a reliable and well-known scale for perceived recovery [18].

The CEQ consists of six questions, with treatment credibility evaluated by items 1-3 and treatment expectancy by items 4-6. Item 4 and item 6 are scored on an 11-point scale from 0% to 100%, while other items are scored on an 8-point scale from 1 to 9. Scores for item 4 and item 6 were transformed to an 8-point scale (1-9), combining 40%, 50%, and 60% into one category, based on previous studies [22,23]. Finally, higher scores indicate higher treatment credibility or expectancy. These transformed 1-9 scores were used in the statistical analysis.

Analysis

Statistical analysis was performed using IBM SPSS Statistics for Windows, Version 28 (Released 2021; IBM Corp., Armonk, New York, United States), except for the bootstrap analysis that was conducted using Excel. Exploratory factor analysis was conducted with the maximum likelihood method and direct oblimin rotation as recommended in a previous study [24]. Factor solutions with eigenvalues >1 were investigated. Additionally, the Kaiser-Meyer-Olkin measure was calculated, and Bartlett’s sphericity test was conducted with the acceptance criteria of a Kaiser-Meyer-Olkin measure >0.5 with a p-value of <0.05.

Cronbach’s alpha was then calculated for each factor identified in the exploratory factor analysis, across all items, across items 1-3 for the credibility subscale proposed by Devilly and Borkovec [6], and across item 4-6 for the expectancy subscale proposed by Devilly and Borkovec [6]. The criterion for acceptable internal consistency was an alpha value of ≥0.7 [13].

Construct validity was examined with convergent validity, where Pearson’s correlation coefficient r with 95% confidence intervals (CIs) of each factor sum score to the PSFS 2.0 change scores and the GRCS were calculated. Satisfactory convergent validity was assumed when the r values ranged from 0.4 to 0.7 [12].

In the CEQ, the main difference between item 2 for the credibility subscale and item 5 for the expectancy subscale is the expression “think” and “feel,” and there is a concern that they are evaluating the same construct [6]. Therefore, we conducted a pairwise comparison to investigate whether there was a difference in item scores. In this study, the sample size was not estimated for testing differences, and thus a type I error could occur; therefore, the comparison was conducted using the bootstrap method, where data were resampled enough to reduce the possibility of the type 1 error while maintaining the variability of the population data. Following previous studies [25,26], 120 resamples were taken and interval estimates were made using four methods: (1) the normal method, (2) the basic method, (3) the studentized method, and (4) the percentile method. Following Yoshihara's recommendation [27], the smallest CI from the four bootstrap methods was selected as the final result. No difference in the scores of the two items was assumed when the CIs of the difference included zero.

## Results

**Figure 1:**
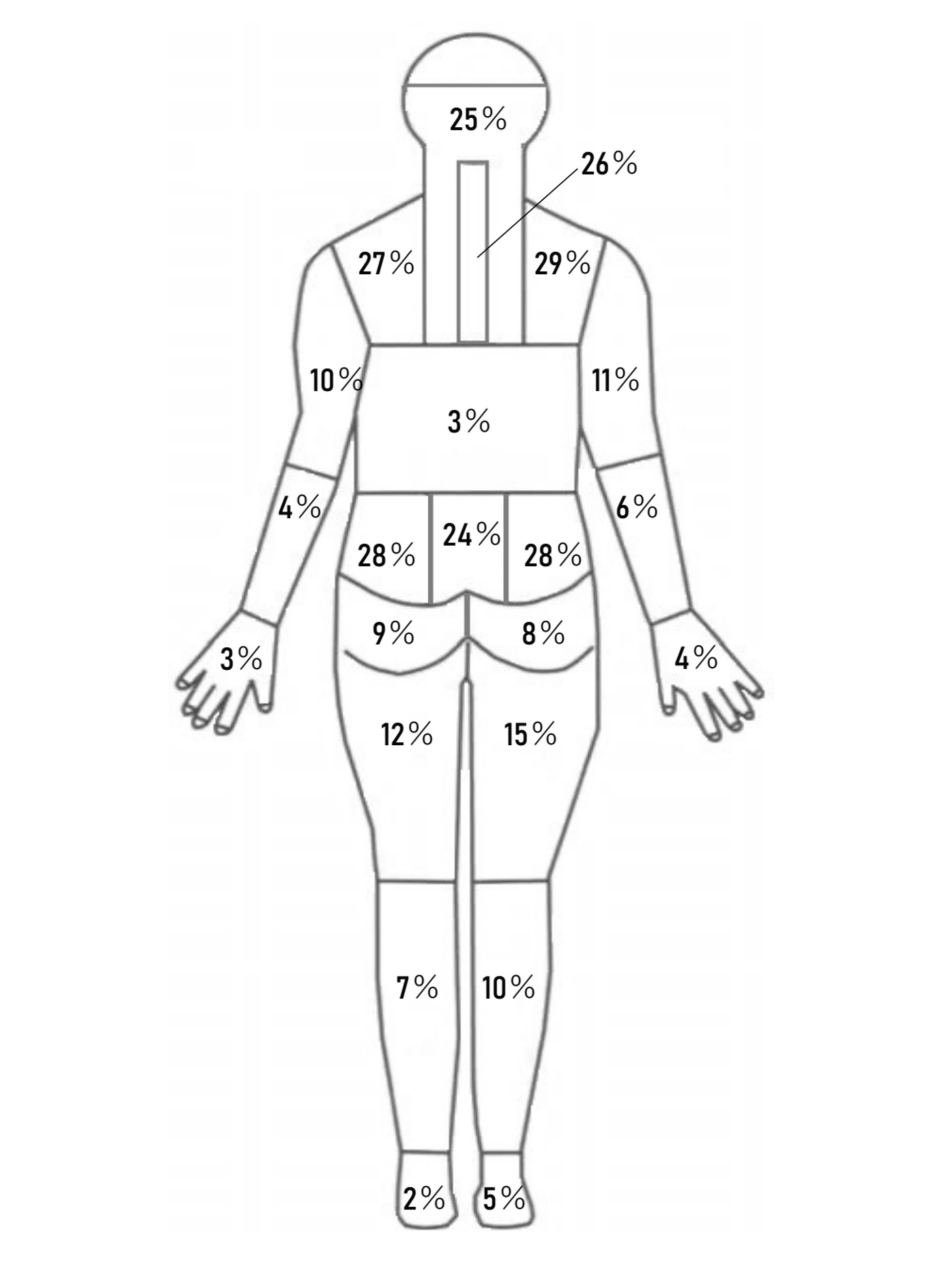
Symptom locations of the participants (N = 100).

Characteristics of the 100 participants and scores of each measure were summarized in Table 1. Symptom locations are summarized in Figure 1. The mean (SD) interval between the baseline and follow-up sessions was 36.8 (22.9) days.

**Table 1:** Characteristics of the participants (N = 100). Values are presented with mean (standard deviations) unless specified.

Variable	Value
Sex	
Number of men	43
Number of women	57
Age (years)	49.25 (18.1)
Symptom duration	
Number of those with less than seven days	20
Number of those with eight days to three months	38
Number of those with greater than or equal to three months	42
Patient-Specific Functional Scale 2.0 at the initial session (0 to 10)	5.9 (2.1)
Patient-Specific Functional Scale 2.0 at the follow-up session (0 to 10)	2.3 (2.1)
Global rating of change scale (-5 to 5)	3.2 (1.2)
Credibility Expectancy Questionnaire total score (6 to 54)	46.4 (6.7)
Credibility Expectancy Questionnaire Credibility subscale score (3 to 27)	23.5 (3.6)
Credibility Expectancy Questionnaire Credibility subscale score (3 to 27)	22.9 (3.6)

Exploratory factor analysis revealed a one-factor structure (Table 2) (Kaiser-Meyer-Olkin measure = 0.87, p < 0.001 in Bartlett’s sphericity test, and percentage of variance for the extraction sums of squared loadings = 62.8%). Cronbach’s alpha was 0.89 across all items, 0.91 for the original credibility subscale, and 0.75 for the original expectancy subscale, all indicating acceptable internal consistency. As post-hoc, an exploration was conducted to see if removing items increased Cronbach's alpha, and the increase in Cronbach's alpha stopped when item 4 was removed following item 6, with an alpha value of 0.93 for the four items.

**Table 2:** Factor loading.

Item description	Factor loading
Item 2. How successfully do you think this treatment will be in reducing your symptoms	0.92
Item 1. How logical does the therapy offered to you seem	0.88
Item 5. How much do you really feel that therapy will help you to reduce your symptoms	0.88
Item 3. How confident would you be in recommending this treatment to a friend	0.85
Item 4. How much improvement in your symptoms do you think will occur	0.62
Item 6. How much improvement in your symptoms do you really feel will occur	0.48

Table 3 presents correlations between CEQ factor scores, the PSFS 2.0 change scores, and the GRCS. Total CEQ scores and each original subscale score satisfied convergent validity with the PSFS 2.0 change scores and the GRCS.

**Table 3:** Pearson’s r-value with 95% confidence intervals comparing the total and each subscale scores of the Credibility Expectancy Questionnaire (CEQ) to the Patient Specific Functional Scale 2.0 (PSFS 2.0) change scores and the global rating of change scale (GRCS). ^*^: scores were transformed to show that positive values indicate improvement; ^†^: p < 0.001

CEQ	PSFS 2.0 change score*	GRCS
Total score	0.48 (0.32 to 0.62)^†^	0.59 (0.45 to 0.71)^†^
Credibility subscale	0.48 (0.31 to 0.62)^†^	0.49 (0.32 to 0.63)^†^
Expectancy subscale	0.43 (0.25 to 0.58)^†^	0.62 (0.48 to 0.73)^†^

Regarding the comparison of item 2 and item 5, the smallest CIs ranged from -0.05 to 0.25 in the studentized method, indicating no difference in the score between the two items.

## Discussion

To the best of the authors' knowledge, this study is the first to examine the internal structure of the CEQ in patients with musculoskeletal disorders undergoing physical therapy. Unlike the two-factor structure of the CEQ in patients receiving psychotherapy [6,8], a one-factor structure was found in this study cohort receiving physical therapy due to musculoskeletal conditions, with no distinction between credibility and expectancy. The lack of difference in scores on item 2, a credibility item, and item 4, an expectancy item, unlike the results of a previous study [6] in patients who received psychotherapy, also suggests that the CEQ has a one-factor structure in the present cohort. On the other hand, sufficient internal consistency was confirmed not only in total but also in each of the subscales. Therefore, when the CEQ is used in the field of musculoskeletal physical therapy, it may be used as a tool with a one-factor structure of predicted treatment outcome expectation although it cannot be prohibited from using scores on either subscale. It should be noted, however, that the credibility subscale would not simply substitute for the CEQ total score, considering that the increase in Cronbach's alpha stopped when only item 6 and item 4 were removed.

The reason why the CEQ was a one-factor structure in this study could seem as both a limitation and a strength, but it is possible that the physical therapy intervention was in line with the principles of the MDT. Although MDT practitioners may differ from general physical therapists in that they focus more on biopsychosocial aspects [28], MDT involves the patient in treatment decisions in accordance with optimal musculoskeletal management [1]. MDT also involves a discussion between therapists and patients about predicted expectations in advance, which is important in shared decision making [29]. In addition, the process of working with the patient to see what will happen and how he or she will respond has common elements with another effective physical therapy approach [30] as it acts as a behavioral experiment that breaks down the patient's assumptions. Thus, it is possible that the gap between what patients feel and what they think they will happen in the future may diminish while they are receiving MDT. We believe that the gap between feeling and thinking about what will happen in the future would not be large in an optimal musculoskeletal physical therapy management because a variety of techniques related to shared decision making and patient-centered care are introduced to change the patient's behavior [1]. Therefore, we believe that the results of this study are applicable to providing quality physical therapy.

In order to promote a patient-centered approach in the field of musculoskeletal physical therapy in the future, it will be necessary to consider not only the predicted treatment outcome expectation but also various patient expectations [5,23] in selecting and modifying treatment. Although this study indicates that the CEQ has the potential to be used as an assessment tool for one of these components, the development of a multidimensional, comprehensive, and clinically friendly assessment tool will be necessary in the future [2,5,23].

Several limitations exist in this study. First, the use of the convenience sampling method in this anonymous survey may have caused a bias in CEQ scores due to the presence of patient self-selection bias. Second, this study verified the convergent validity of the CEQ by testing and confirming the correlation between PSFS 2.0 which has high responsiveness to treatment in the field of musculoskeletal physical therapy [21] and GRCS which is widely used; however, divergent validity has not been verified. Therefore, further verification of construct validity is required.

## Conclusions

This study revealed a single-factor loading of the CEQ among patients with musculoskeletal disorders. Additionally, the construct validity of the CEQ was partially identified with convergent validity. Future studies are warranted to develop an optimal tool to measure the patient’s predicted treatment outcome expectations to facilitate the implementation of the patient-centered approach.

## References

[1] Lin I, Wiles L, Waller R (2020). What does best practice care for musculoskeletal pain look like? Eleven consistent recommendations from high-quality clinical practice guidelines: systematic review. Br J Sports Med.

[2] Ebrahim S, Malachowski C, Kamal El Din M, Mulla SM, Montoya L, Bance S, Busse JW (2015). Measures of patients' expectations about recovery: a systematic review. J Occup Rehabil.

[3] Mondloch MV, Cole DC, Frank JW (2001). Does how you do depend on how you think you'll do? A systematic review of the evidence for a relation between patients' recovery expectations and health outcomes. CMAJ.

[4] Laferton JA, Kube T, Salzmann S, Auer CJ, Shedden-Mora MC (2017). Patients’ expectations regarding medical treatment: a critical review of concepts and their assessment. Front Psychol.

[5] Bialosky JE, Bishop MD, Cleland JA (2010). Individual expectation: an overlooked, but pertinent, factor in the treatment of individuals experiencing musculoskeletal pain. Phys Ther.

[6] Devilly GJ, Borkovec TD (2000). Psychometric properties of the credibility/expectancy questionnaire. J Behav Ther Exp Psychiatry.

[7] Kazdin AE (1979). Therapy outcome questions requiring control of credibility and treatment-generated expectancies. Behav Ther.

[8] Silva S, Barbosa E, Salgado J, Cunha C (2021). Portuguese validation of the credibility/expectancy questionnaire in routine practice. Res Psychother.

[9] Tilbury C, Haanstra TM, Verdegaal SH, Nelissen RG, de Vet HC, Vliet Vlieland TP, Ostelo RW (2018). Patients' pre-operative general and specific outcome expectations predict postoperative pain and function after total knee and total hip arthroplasties. Scand J Pain.

[10] Smith MD, Vuvan V, Collins NJ, Hunter DJ, Costa N, Smith MM, Vicenzino B (2023). Protocol for a randomised feasibility trial comparing a combined program of education and exercise versus general advice for ankle osteoarthritis. J Foot Ankle Res.

[11] Takasaki H, Ishida S (2024). Confirmation of the unidimensionality of the satisfaction and recovery index among those with various musculoskeletal disorders. Cureus.

[12] Takasaki H (2022). Cross-cultural adaptation of the satisfaction and Recovery Index among Japanese people with musculoskeletal disorders. J Phys Ther Sci.

[13] Mokkink LB, Prinsen C, Patrick DL (2024). COSMIN methodology for systematic reviews of patient-reported outcome measures (PROMs). COSMIN methodology for systematic reviews of patient-reported outcome measures.

[14] Takasaki H (2023). Predictors of 1-year perceived recovery, absenteeism, and expenses due to low back pain in workers receiving mechanical diagnosis and therapy: a prospective cohort study. Healthcare (Basel).

[15] Ishigaya Y, Kikkawa K, Handa Y (2022). Cross-cultural adaptation of the patient specific functional scale 2.0. Toshurigakuryouhou.

[16] Thoomes-de Graaf M, Fernández-De-Las-Peñas C, Cleland JA (2020). The content and construct validity of the modified patient specific functional scale (PSFS 2.0) in individuals with neck pain. J Man Manip Ther.

[17] Cleland JA, Fritz JM, Whitman JM, Palmer JA (2006). The reliability and construct validity of the neck disability index and patient specific functional scale in patients with cervical radiculopathy. Spine (Phila Pa 1976).

[18] Kamper SJ, Maher CG, Mackay G (2009). Global rating of change scales: a review of strengths and weaknesses and considerations for design. J Man Manip Ther.

[19] Takasaki H, Handa Y, Kikkawa K (2024). Mechanical diagnosis and therapy has a clinically meaningful effect on neck derangement syndrome with a directional preference for cervical retraction or extension in comparison to a wait-and-see approach: an assessor-blinded randomized controlled trial for 6 weeks. JOSPT Open.

[20] Ito M, Okumura Y, Horikoshi M (2016). Japan unified protocol clinical trial for depressive and anxiety disorders (JUNP study): study protocol for a randomized controlled trial. BMC Psychiatry.

[21] Stewart M, Maher CG, Refshauge KM, Bogduk N, Nicholas M (2007). Responsiveness of pain and disability measures for chronic whiplash. Spine (Phila Pa 1976).

[22] Smeets RJ, Beelen S, Goossens ME, Schouten EG, Knottnerus JA, Vlaeyen JW (2008). Treatment expectancy and credibility are associated with the outcome of both physical and cognitive-behavioral treatment in chronic low back pain. Clin J Pain.

[23] Haanstra TM, Tilbury C, Kamper SJ (2015). Can optimism, pessimism, hope, treatment credibility and treatment expectancy be distinguished in patients undergoing total hip and total knee arthroplasty?. PLoS One.

[24] Costello A, Osborne JW (2005). Best practices in exploratory factor analysis: four recommendations for getting the most from your analysis. Pract Assess Res Eval.

[25] Takasaki H (2024). Developing a final format of a patient-reported outcome measure for disability in daily living due to stiff neck/shoulders, Katakori disability index, through internal structure assessments. Musculoskelet Care.

[26] Takasaki H, Yanagisawa M (2020). Normative ability of young females to control the lumbopelvic curvature during active knee extension in sitting. J Phys Ther Sci.

[27] Yoshihara K (2009). Data analysis using bootstrap method with Excel [Excelによるブートストラップ法を用いたデータ解析].

[28] Takasaki H, Saiki T, Iwasada Y (2014). McKenzie therapists adhere more to evidence-based guidelines and have a more biopsychosocial perspective on the management of patients with low back pain than general physical therapists in Japan. Open J Ther Rehabil.

[29] Geurts JW, Willems PC, Lockwood C, van Kleef M, Kleijnen J, Dirksen C (2017). Patient expectations for management of chronic non-cancer pain: a systematic review. Health Expect.

[30] Miki T, Kondo Y, Kurakata H, Buzasi E, Takebayashi T, Takasaki H (2022). The effect of cognitive functional therapy for chronic nonspecific low back pain: a systematic review and meta-analysis. Biopsychosoc Med.

